# Complication of Gastric Balloon in an Adolescent Patient: A Case Report

**DOI:** 10.7759/cureus.73743

**Published:** 2024-11-15

**Authors:** Rachel Siretskiy, Dailen Alonso, Juan Calisto, Patricio E Lau

**Affiliations:** 1 Surgery, Florida International University, Herbert Wertheim College of Medicine, Miami, USA; 2 Surgery, Baylor University Medical Center, Waco, USA; 3 Pediatric Surgery, Nicklaus Children's Hospital, Miami, USA

**Keywords:** adolescents, intragastric balloon, medical tourism, obesity, pediatrics

## Abstract

As the prevalence of childhood obesity continues to rise, there is an increase in demand for temporary and minimally invasive alternatives to bariatric surgery as solutions for addressing pediatric obesity. Intragastric balloon (IGB) placement is an increasingly popular methodology for addressing adult obesity; however, it is not approved for the pediatric population.

We describe the case of a 17-year-old adolescent female who underwent IGB placement in Colombia and failed to receive proper follow-up care in the country of insertion resulting in a gastric outlet obstruction. This highlights the need for United States-based pediatric surgical providers to be familiar with IGB procedures as an increase in the popularity of medical tourism creates circumstances in which these providers could be expected to manage associated follow-up care and complications related to IGB placement.

## Introduction

The prevalence of pediatric obesity has been on the rise in recent decades and has become a growing global public health problem [1]. Childhood obesity leads to the development of comorbid psychosocial and health problems such as depression, social withdrawal, suicidal behavior, type 2 diabetes mellitus (T2DM), reduced bone mass, liver dysfunction, and polycystic ovarian syndrome [2].

Additionally, studies show that childhood obesity also leads to long-term repercussions in adulthood such as increased risk of cardiovascular disease, hyperlipidemia, T2DM, joint disease, and obstructive sleep apnea [1]. To better manage growing rates of obesity and reduce secondary co-morbidities from developing, there have been advancements in bariatric surgery to include a variety of treatment options, including the intragastric balloon (IGB).

The IGB has been used for many decades to reduce food intake along with lifestyle modification to achieve weight loss in obese adult individuals. Although the IGB is considered safe in adults, there is very limited data on the safety of this procedure and its complications in adolescents [1]. Epigastric pain, nausea, and vomiting are some of the common side effects reported at the time of balloon placement, but there is no description of long-term complications of the IGB [1-5].

Despite IGB not being approved for pediatric use in the United States (US), the global increase in medical tourism has created opportunities for pediatric patients to obtain these procedures in countries where it is approved, only to return without proper post-operative management which is left in the hands of US-based providers. We describe the case of a 17-year-old female who received an IGB placement in Columbia and developed a post-operative complication after returning to her home in the US.

## Case presentation

A 17-year-old female with a past medical history of depression and suicidal ideation, anxiety, post-traumatic stress disorder (PTSD), bulimia, and gastric balloon placement in Colombia 15 months ago presented to the Emergency Department (ED) with one day of generalized abdominal pain that was worse in the upper quadrants bilaterally. The patient denied current diarrhea, vomiting, and changes in stool color, but endorsed multiple bouts of vomiting, and one loose non-bloody stool one day prior to presentation. Presenting vital signs were a temperature of 98.2F, heart rate of 81, respiratory rate of 18, oxygen saturation of 99%, and blood pressure of 121/60. Pertinent physical examination findings included abdominal tenderness in all four quadrants with moderate distention noted in the right and left upper quadrants. She had normal bowel sounds. Her body mass index (BMI) in 2019 was 31, and after balloon placement, it was 23.

**Figure 1:**
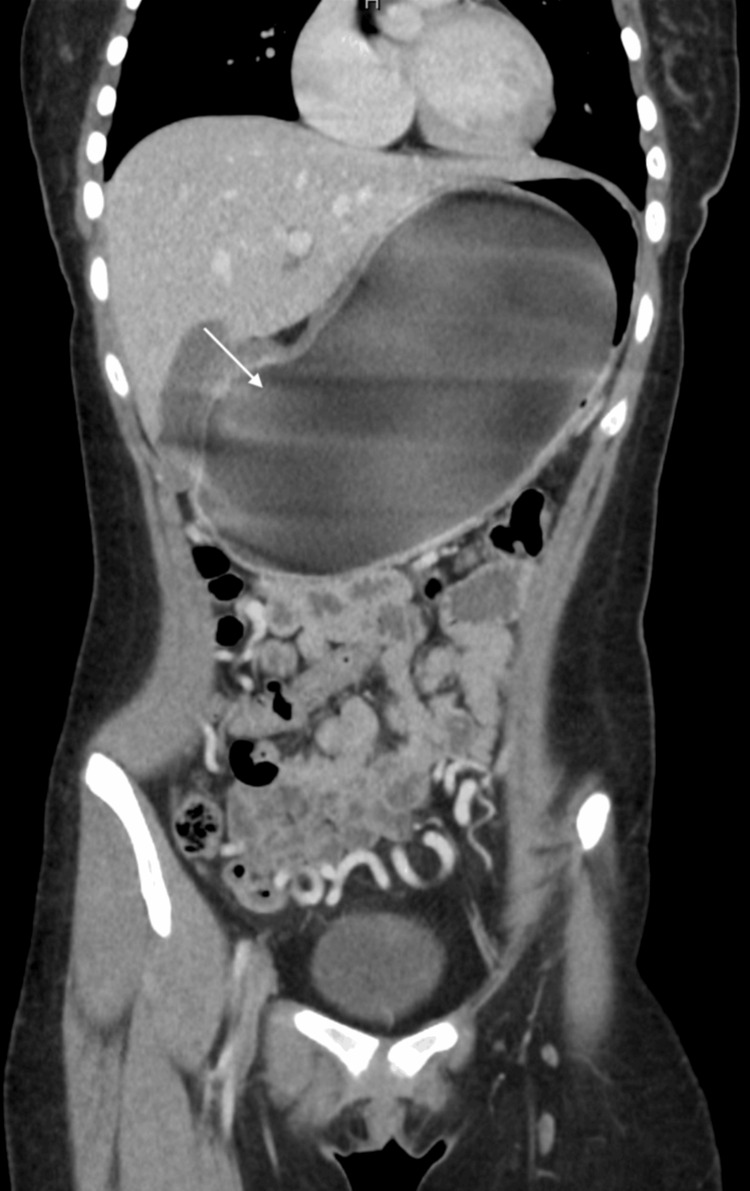
Coronal view of CT scan of patient’s abdomen with the intragastric balloon from referral hospital

**Figure 2:**
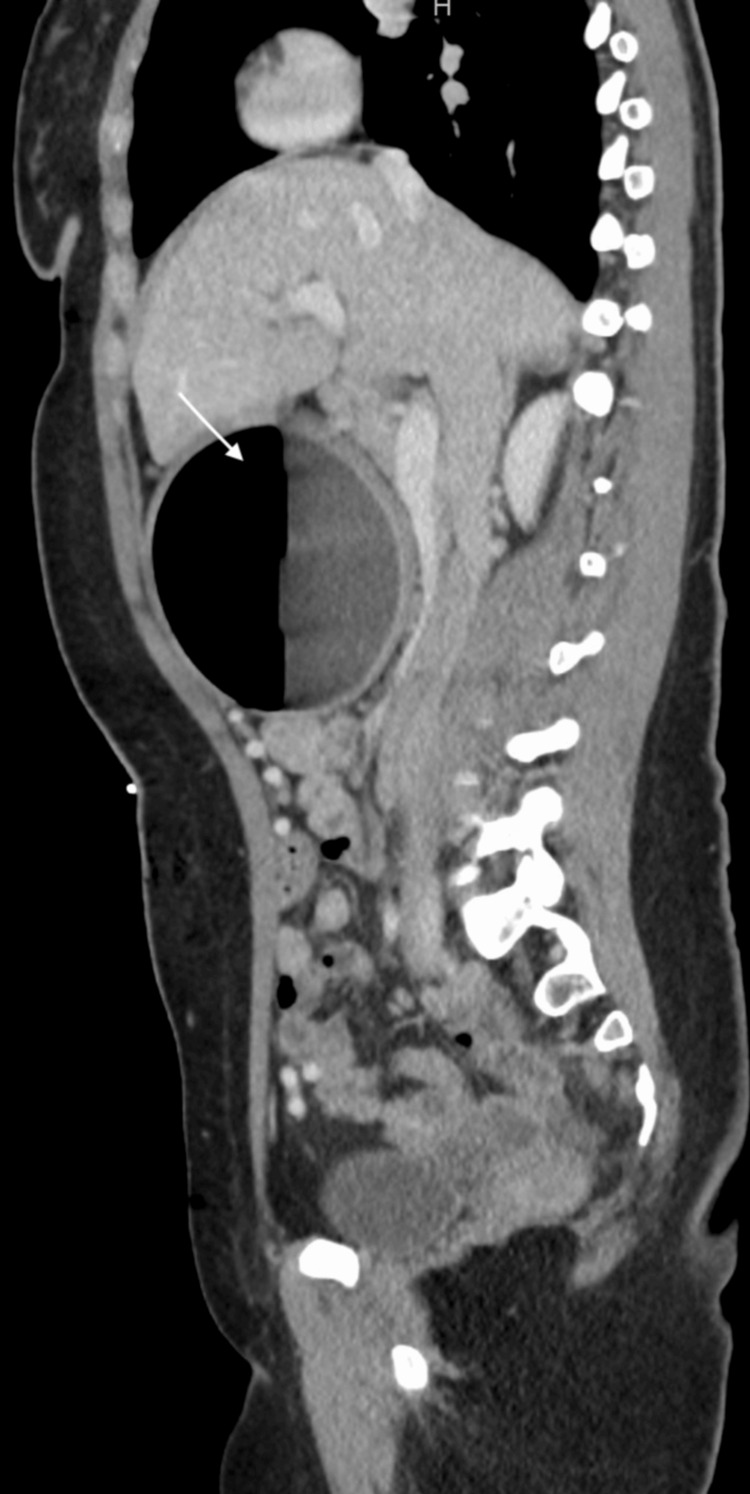
Sagittal view of CT scan of patient’s abdomen with the intragastric balloon from referral hospital

**Figure 3:**
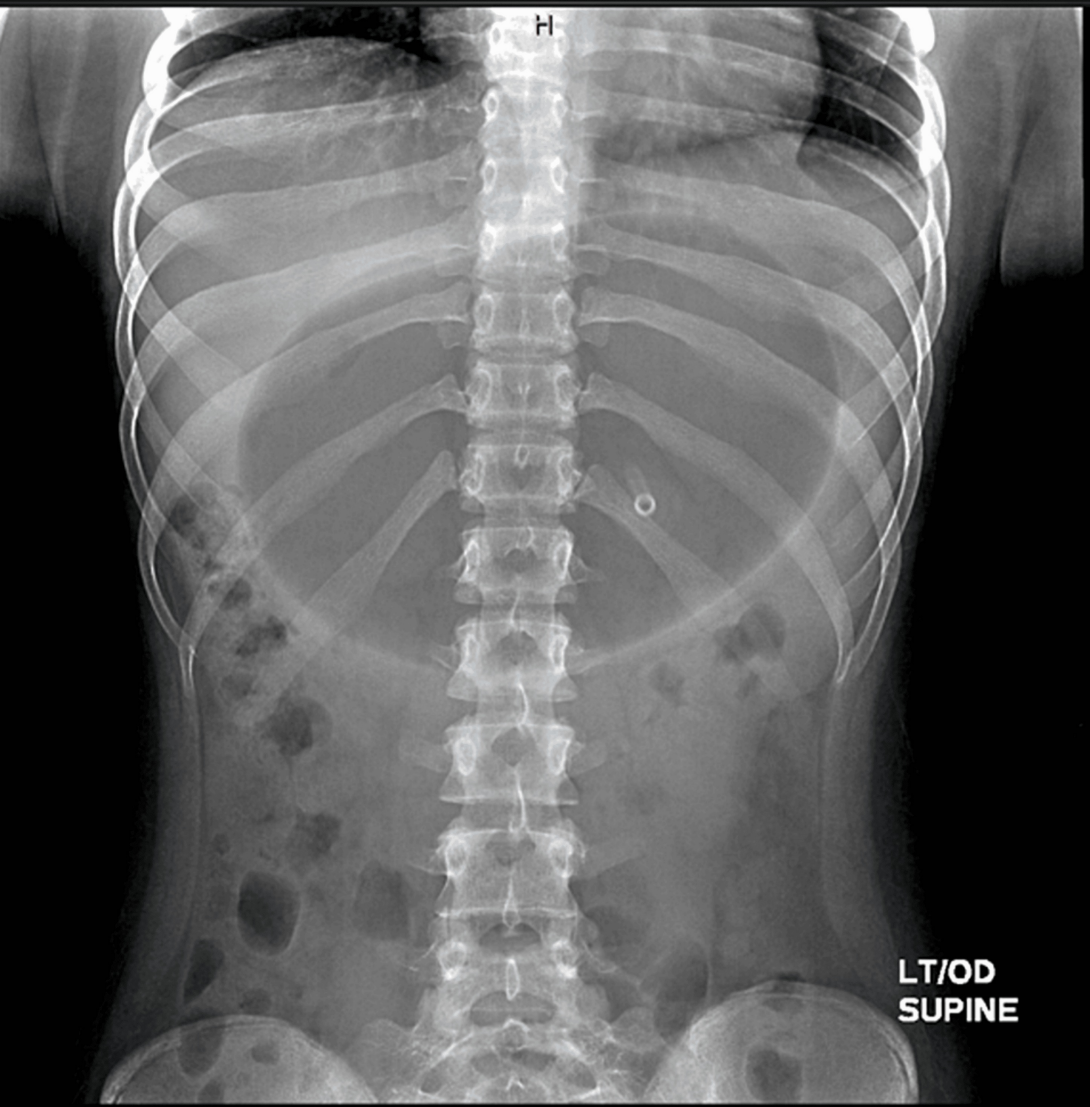
Anterior-posterior x-ray of the intragastric balloon

The patient originally went to an ED from an outside hospital nearby, where a computerized tomography (CT) scan of the abdomen and pelvis was performed and the IGB was visualized (Figures 1, 2). CT scan findings showed extensive gastric distention caused by an intra-luminal balloon. There was partial compression of the splenic and proximal aspect of the superior mesenteric vein (SMV) with significant decompressing mesenteric collaterals extending into the pelvis. There was also a mass effect on the pancreas and third portion of the duodenum leading to proximal duodenal dilatation. There was poor visualization of the inferior vena cava (IVC) so an ultrasound of the IVC was conducted for further evaluation. The patient was then referred to our pediatric facility where IVC Doppler ultrasound findings revealed normal caliber of the IVC without filling defects or collateral formation. An ultrasound of the appendix was also performed to rule out appendicitis and demonstrated a normal appendix and a cluster of prominent veins in the right lower quadrant. A pelvic ultrasound was ordered to further evaluate the patient and yielded multiple dilated and tortuous venous collaterals in the pelvis related to venous compression by a dilated stomach. An anterior-posterior (AP) view x-ray of the abdomen showed gastric distention due to a large intra-luminal balloon, and a non-obstructive bowel gas pattern (Figure 3). The decision was made by the surgical team to remove the IGB since it was causing gastric outlet obstruction and compression of abdominal vasculature.

**Figure 4:**
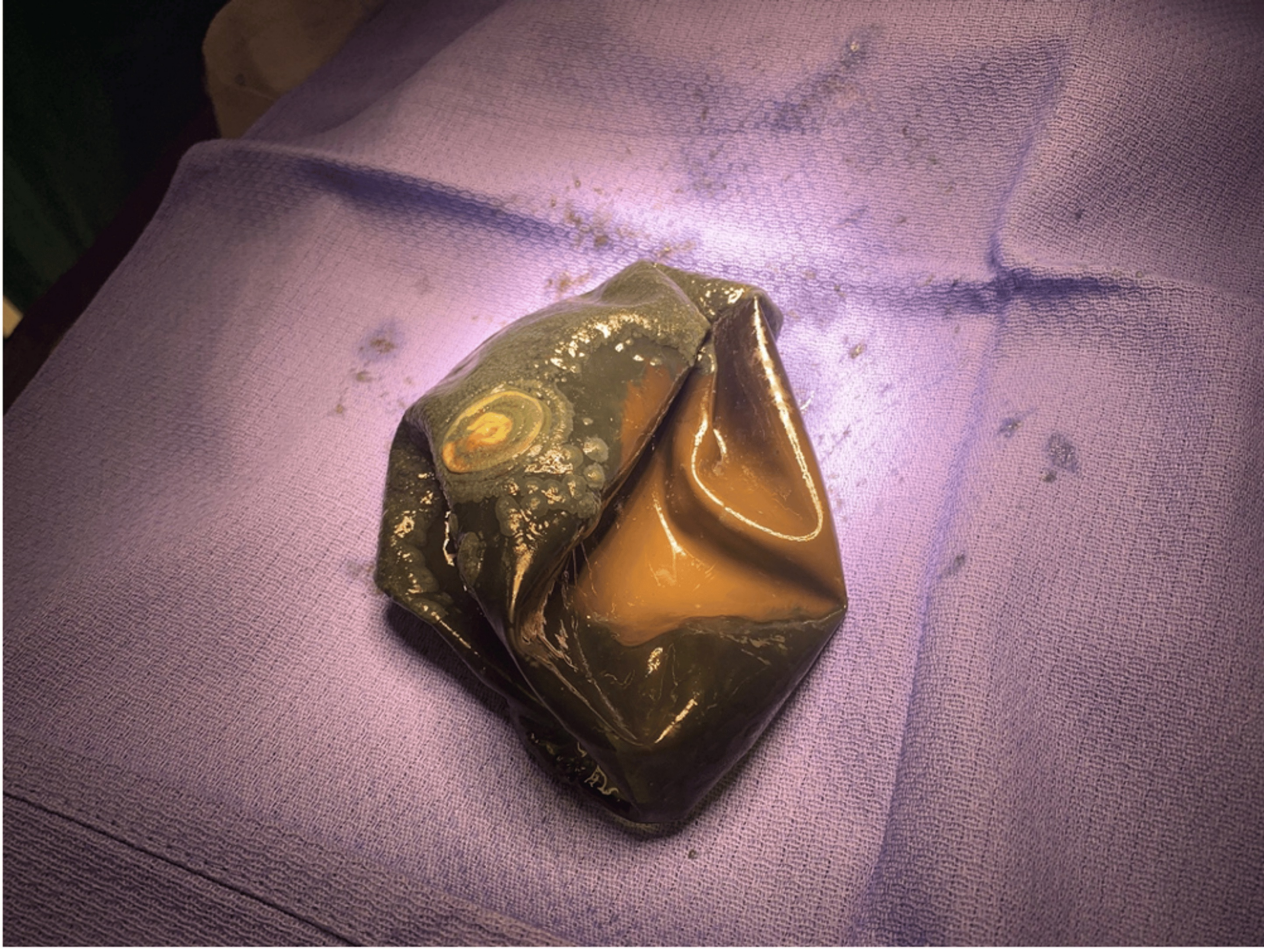
Intragastric balloon after endoscopic removal from the stomach

In the operating room, after successful endotracheal intubation, an esophagogastroduodenoscopy (EGD) scope was inserted, and a large balloon was identified in the stomach. A piercing device was inserted through the EGD scope to puncture through the balloon and desufflate it. After one liter of fluid was suctioned from the stomach, the balloon was successfully removed through the mouth with special graspers that were inserted through the EGD scope (Figure 4). After removing the balloon, a second look at the esophagus and the stomach was performed. The esophagus appeared viable, but there was a significant amount of reflux indicated by the presence of irritation in the lower portion. The stomach was viable with no areas of necrosis or injury. The patient was then extubated and taken to the post-anesthesia care unit for observation and recovery. She was started on a regular diet the same day and discharged the following day.

## Discussion

Intra-gastric balloons have been used as an adjunct weight loss method for adults since the 1980s. This is a minimally invasive and temporary procedure that involves the placement of a saline or air-filled balloon endoscopically. This reduces the volume of the gastric cavity and induces a sensation of satiety while also demonstrating the properties of reducing gastric emptying. It is most effective when used in conjunction with lifestyle modifications [6-8]. There is concern with the utility of IGBs as they have the potential to cause fatal secondary events such as gastric perforation, gastric ulceration, and intestinal obstruction [8]. Despite IGB devices showing benefits for weight reduction and management of obesity in adults, it has not been recommended for use in pediatric populations because of limited data that describes the long-term utility, outcomes, and complications of IGB in pediatric populations. As of now, there are limited studies on the utility and complications of IGB in pediatric populations. Conclusions within these studies remain mixed with some demonstrating that IGB led to weight loss initially and reduction in associated co-morbidities, but others concluding that there was no benefit when it comes to long-term weight management in adolescent populations [6,7]**.**

However, medical tourism, defined as international travel in the pursuit of medical care, continues to expand as a source for patients to obtain procedures not indicated in the US, as seen in the case described [9]. In 2020, the largest medical tourism market in the US was estimated at $18 billion, and this figure was projected to reach US $31.2 billion by 2027 [10]. Per the Centers for Disease Control, the most common countries frequented by US residents are Argentina, Brazil, Canada, Colombia, Costa Rica, Cuba, the Dominican Republic, Ecuador, Germany, India, Malaysia, Mexico, Nicaragua, Peru, Singapore, and Thailand [11]. US medical tourists pursue cancer treatment, dental care, fertility treatments, organ and tissue transplantation, and various forms of surgery, including bariatric, cosmetic, and non-cosmetic [11]. Despite the lack of evidence supporting IGB as a treatment for childhood obesity, pediatric populations still obtain the treatment through medical tourism.

This case highlights these circumstances as it describes a pediatric patient who underwent placement of IGB in Colombia without removal then necessitated intervention at a US facility. This serves as a unique assessment of pediatric presentations and complications that might present themselves secondary to US-based pediatric individuals pursuing these procedures in foreign countries and then failing to have follow-up care. It also highlights a need to examine potential complications and courses of management of such procedures particularly among the pediatric populations. This will guarantee that providers are better equipped to anticipate avenues of management since more people are traveling to other countries to receive procedures not indicated in the US.

Particularly in the scope of this case, follow-up care for pediatric patients who underwent IGB placement in foreign countries should focus on confirmation that the balloon was removed following an appropriate timeline. Literature highlights that the typical practice is to remove intra-gastric balloons six months after insertion. Studies have shown that balloons retained for more than six months were associated with higher rates of balloon rupture, displacement, and intestinal obstruction [12]. Cases have even been reported with prolonged retention of the balloon leading to migration and subsequent gastric outlet obstruction as a result which could have fatal implications [13-16]. Therefore, pediatric surgery providers must be familiar with IGB procedures in instances that like in this case there is a need for intervention by these providers.

Limitations of these studies are that they all contain small sample sizes, have a pre-dominance to towards having a female study population, and have very short follow-up periods that make it difficult to make conclusions about long-term results. Therefore, this highlights a need for further exploration of IGB as a potential modality for addressing pediatric and adolescent obesity as it is reversible and less invasive than its other bariatric counterparts.

The strength of this report is that it identifies a potential surgical presentation that may arise secondary to increased medical tourism and access to bariatric procedures that are not approved in the US. This provides general guidance on how to best manage this presentation in a pediatric patient. The limitations of this report are that generalizability may be limited, particularly as it applies to centers that do not see high volumes of immigrant patients.

## Conclusions

Increased global application of IGB as a method for managing childhood obesity demonstrates a need for pediatric surgeons based in the US to be more aware of potential complications and avenues for management. This is particularly true for balloon removal which is to occur more than six months after placement and can be overlooked when patients travel to other countries temporarily for placement. Further, there is a need for additional studies that examine the efficacy of IGBs as a potential mechanism for addressing pediatric obesity in the US. 
